# The Inuit gut microbiome is dynamic over time and shaped by traditional foods

**DOI:** 10.1186/s40168-017-0370-7

**Published:** 2017-11-16

**Authors:** Geneviève Dubois, Catherine Girard, François-Joseph Lapointe, B. Jesse Shapiro

**Affiliations:** 1 Département de sciences biologiques, Université de Montréal, 90 Vincent-d’Indy, Montréal, Qc H2V2S9 Canada; 2 Center for Northern Studies, Département de sciences biologiques, Université de Montréal, 90 Vincent-d’Indy, Montréal, Qc H2V2S9 Canada

**Keywords:** Gut microbiome, Inuit traditional diet, Temporal variation, Western diet, Dietary transition, 16S rRNA gene

## Abstract

**Background:**

The human gut microbiome represents a diverse microbial community that varies across individuals and populations, and is influenced by factors such as host genetics and lifestyle. Diet is a major force shaping the gut microbiome, and the effects of dietary choices on microbiome composition are well documented. However, it remains poorly known how natural temporal variation in diet can affect the microbiome. The traditional Inuit diet is primarily based on animal products, which are thought to vary seasonally according to prey availability. We previously investigated the Inuit gut microbiome sampled at a single time point, and found no detectable differences in overall microbiome community composition attributable to the traditional Inuit diet.

**Results:**

To determine whether seasonal changes in the Inuit diet might induce more pronounced changes in the microbiome, we collected stool and toilet paper samples, and dietary information from Inuit volunteers living in Resolute Bay (Nunavut, Canada), and compared them to individuals of European descent living in Montréal (Québec, Canada) consuming a typical Western diet. We sequenced the V4 region of the 16S rRNA gene to characterize microbiome diversity and composition, and compared samples collected with toilet paper or from stool. Our results show that these sampling methods provide similar, but non-identical portraits of the microbiome. Based on toilet paper samples, we found that much of the variation in microbiome community composition could be explained by individual identity (45–61% of variation explained, depending on the beta diversity metric used), with small but significant variation (3–5%) explained by sex or geography (Nunavut or Montréal). In contrast with our previous study at one time point, sampling over the course of a year revealed that diet explains 11% of variation in community composition across all participants, and 17% of variation specifically among Nunavut participants. However, we observed no clear seasonal shifts in the microbiomes of participants from either Nunavut or Montréal. Within-individual microbial diversity fluctuated more over time in Nunavut than in Montréal, consistent with a more variable and highly individualized diet in Nunavut.

**Conclusions:**

Together, these results shows that the traditional Inuit diet and lifestyle has an impact on the composition, diversity and stability of the Inuit gut microbiome, even if the seasonality of the diet is less pronounced than expected, perhaps due to an increasingly westernized diet.

**Electronic supplementary material:**

The online version of this article (10.1186/s40168-017-0370-7) contains supplementary material, which is available to authorized users.

## Background

The human gut microbiome is a diverse community of microbial eukaryotes, viruses, archaea, and mostly bacteria [1–3], many of which play important roles in immunity, metabolism and nutrition [4–6]. The community structure of the microbiome is determined by many factors, including geography, sex, host genetics, and age [7–11].

Microbiome composition and structure may also vary within individuals over time, although most individuals have a relatively stable microbiome [12, 13]. Individuals with highly diverse microbiomes tend to be more stable through time [12]. Other studies have shown that individuality is preserved through time, underlying an overall stable and personalized microbiome [14]. On time scales of days to weeks, diet is the main factor driving composition and structure of the gut microbiome [13]. For example, some types of dietary fiber transits through the digestive tract without being assimilated by the human body, providing a food source for fermentative bacteria [15]. High fiber intake favors the presence of several members of the phylum *Bacteroidetes*, which can break down complex carbohydrates [16]. Impacts of fat intake on the gut microbiome are less well known. However, bile tolerant taxa such as *Alistipes*, *Bilophila*, and *Bacteroides* increased in relative abundance after a week of high fat and protein consumption, while *Firmicutes* decreased [17].

Traditional diets vary widely across human populations. In a pioneering study, the microbiomes of children from Burkina Faso, consuming a carbohydrate-based traditional diet, were compared to the microbiomes of Italian children eating a westernized diet containing more animal fats and proteins [18]. *Bacteroidetes* (particularly *Prevotella*) were more prevalent among Burkinabés while *Firmicutes* were prevalent among Italians. The Italian gut microbiome was also compared to hunters-gatherers from the Hadza community in Tanzania [19]. Significantly higher microbial diversity was observed in the Hadza, along with enrichment of *Prevotella* and *Treponema*, possibly allowing more efficient fiber fermentation in the gut. A study also showed significant differences between rural Malawians, Venezualans, and Americans, with an under-representation of *Prevotella* in Americans [9].

Although diet is known to alter microbiome composition over time scales of days to weeks [13], few studies have followed seasonally variable diets and their impacts on the microbiome. One exception is a study, which looked at temporal changes in the microbiome in the Hutterite Anabaptist community (USA), in which traditional diet relies on seasonal food availability [20]. In particular, significant amounts of fruits and vegetables are only available during the summer. This study revealed a significant shift in species composition, as well as a lower diversity in the summer compared to winter. The increased consumption of fibrous fruits and vegetables during summer could be responsible for these differences, favoring blooms of bacteria specialized in fiber digestion.

The Inuit of the Canadian Arctic have traditionally been hunter-gatherers [21]. The Inuit traditionally consume an animal-rich diet, composed of marine and terrestrial mammals (e.g., seal and caribou), as well as wild birds and fish. Meat and fish can be consumed raw, frozen, cooked, or fermented [22]. Several types of plants and berries (e.g., blueberries, blackberries, cranberries) are also consumed [22], but the majority (75%) of calories come from animal fat [23]. In contrast, Montrealers get ~35% of their calories from fat and ~50% from carbohydrates. Differences between English and French speakers are negligible, suggesting that Montrealers are fairly representative of the North American diet [24]. The traditional Inuit diet is rich in proteins and essential vitamins, and also contributes to social bonding and cultural preservation [25]. However, with increasing access to imported supermarket foods, Inuit populations are currently experiencing a transition toward a more Western diet [26, 27]. Global warming, ice thinning, and changing prey migratory patterns also contribute to this transition. Therefore, the Inuit diet is now a mixture of traditional and Western foods, depending on the individual and the community, modulated by food insecurity [28, 29].

In the Canadian territory of Nunavut, the Inuit traditionally divide their year into six seasons [30] (Additional file 1 Table S1) characterized by different activities and land use. For example, open-water hunting trips occur more frequently from June to November, while land-based trips occur from December to May, potentially affecting diet according to prey availability [31]. Seasonal variation in nutrient intake from both traditional and market food has been observed in Inuit communities from Baffin Island (Nunavut) [32]. Over a year-long survey, traditional foods were consumed most often in August and September, whereas market foods were most popular in October and November. Significant seasonal variations in energy intake have been observed for traditional food, but not for market food [32]. Food security is an issue in many Nunavut communities (particularly for women who often save the highest quality foods for their children) and is the outcome of many determinants (e.g., store food availability, high hunting cost, climate changes etc.) [33].

We recently characterized the gut microbiomes of 19 Inuit individuals, and compared them to 26 Montrealers consuming a typical Western diet [34]. We showed that Inuit and Montréal gut microbial communities were generally similar in terms of composition and diversity, possibly owing to the increasing prevalence of Western diets in the North. Gut microbial community composition or diversity was not significantly affected by age, sex, or body mass index (BMI)—although Inuit did have higher BMI on average [34]. However, the study was based on a single time point, taken in the summer. It is possible that the Inuit and Western diets (and the corresponding microbiomes) are more distinct at other times of the year, due to seasonal variation in the availability of both traditional and store-bought foods.

To investigate the temporal dynamics of the Inuit gut microbiome, we collected approximately monthly microbiome samples from Inuit volunteers from a community in Nunavut (Canada), and compared them to a control group from Montréal (Canada), consuming a typical Western diet. We used deep amplicon sequencing of the V4 region of the 16S marker gene to assess the diversity and composition of the gut microbiome. We hypothesized that Westerners consume a fairly stable diet over time, due to yearlong availability of a variety of foods at the supermarket, which we expected would result in a relatively stable microbiome. In contrast, we hypothesized that Inuit microbiomes would be more variable over time, due to seasonal availability of different traditional foods. For ease of sample collection and shipping from a remote location, study participants used toilet paper to sample their own stool. This convenient sampling method has been used previously [12] but the differences in microbiome surveys between toilet paper and whole stool have not yet been characterized. Here, we show that toilet paper and whole stool provide similar, but non-identical portraits of the microbiome. Based on sequencing these toilet paper samples, we found that microbiome community composition varies within individuals, but that there is greater variation between individuals than within an individual over time, both in the Nunavut and Montréal cohorts. Diet differed markedly between Montréal and Nunavut, and explained a significant amount of the variation in microbiome composition. Within-individual temporal variation was higher in Nunavut than in Montréal, suggesting a more variable microbiome, possibly due to a more variable diet. However, no seasonal or monthly shift of microbiome composition was detected in either Nunavut or Montréal over the time scale sampled.

## Methods

### Participant recruitment and sample collection

Resolute Bay, Nunavut, is the second northernmost community in Canada (Additional file 2 Figure S1). We recruited 15 Inuit participants between 24 and 67 years old (mean of 46.5 years old) from this small hamlet (approximate population: 215 inhabitants, mostly of Inuit descent [35]). Nine non-Inuit participants of European descent living in Montréal, Canada, between 23 and 48 years old (mean of 30.2 years old), and mostly from a university community, were also recruited for comparison. Each participant provided a paired stool sample and toilet paper containing stool from the same bowel movement at the beginning of the study, for methodological comparisons. Subsequent samples were taken once per month, using toilet paper only. Samples were collected from July 2015 to February 2016 and also in July 2016 in Resolute Bay (details in Additional file 1 Table S1), and from October 2015 to May 2016 in Montréal. Each sample was accompanied by a dietary habit questionnaire containing information about food consumption in the 48 h preceding the sampling event (Additional file 3 File 1). The questionnaire recorded the number of times (frequency) particular categories of food were eaten in the past 48 h, but did not quantify the amount of food consumed. During the sample collection, participants wore sterile gloves and used sterile toilet paper. In Nunavut, when the sampling coordinator was present (July 2015 and July 2016), the samples were kept outside (temperature < 4 °C) for a maximum of 12 h before being collected and frozen at − 80 °C until DNA was extracted. During the rest of the year, a local resident was hired as a sampling assistant. During this period, toilet paper samples were kept outside (temperature between − 35 and 0 °C) for a maximum of 2 days and were shipped along with self-reported questionnaires by mail, without additional preservation methods (at ambient temperature). Shipment duration varied between 5 and 7 days. Upon reception in the lab, the samples were kept frozen at − 80 °C until DNA extraction. For sampling in Montréal, samples were kept in a refrigerator for a maximum of 12 h before being frozen at − 80 °C. Therefore, Nunavut samples from July were kept under “optimal preservation” conditions while samples from other months were kept in “sub-optimal preservation” conditions. The possible influences of those differences are considered in the discussion.

### DNA extraction, library preparation, and sequencing

Prior to all laboratory work, each sample was assigned an anonymized number and was processed in a random order and random location in 96-well plates to avoid confounding batch effects and temporal variation in the data. DNA was extracted from stool and toilet paper samples with PowerSoil® DNA isolation kit (MO BIO Laboratories) using the stool sample protocol provided with the kit for both types of samples. A sample-free tube was used as a negative control in the extraction process. DNA concentration for each sample was measured using Qubit® 2.0 Fluorometer (Life Technologies) and then normalized to 5 ng/μL. No DNA was detected in extraction blanks. The library preparation was performed by two-step polymerase chain reaction (PCR) method. The V4 region of the 16S ribosomal RNA gene segment was amplified in a first step PCR reaction (Step 1), set up in 25 μL volumes, each containing 5 ng/μL of template DNA, 1× of 5X Phusion HF, 1 mmol/L of each dNTP, 3 μmol of each primer (Additional file 1 Table S2), Phusion® High-Fidelity DNA Polymerase (Life Technologies) and 9.25 μL of sterile H_2_O. Amplifications were performed in a Eppendorf® Mastercycler® nexus thermal cycler (Fisher Scientific), programmed with an initial denaturation step at 98 °C for 30 s, followed by 20 cycles at 98 °C for 25 s, 54 °C for 40 s, 72 °C for 30 s and a final elongation step at 72 °C for 2 min. PCR quadruplicates were performed for each sample, which were then pooled and purified using Agencourt AMPure XP (Beckman Coulter). PCR products of the expected size (350–360 bp) were verified using the QIAxel® Advanced System (QIAGEN) with the method 0 M500, using 10 s injections with a 30 ng/μL marker. A second PCR reaction was performed to attach 9 bp sequencing barcodes and Illumina® adapter sequences to each sample, using step 1 PCR purified products as template DNA. Step 2 PCR was carried out in 25 μL volume, using the same reagent concentrations as for step 1 PCR, with 4 μL of Step 1 PCR products and specific primers for this step (Additional file 1 Table S2). Amplifications were performed with an initial denaturation step at 98 °C for 30s and 7 cycles of amplification at 98 °C for 30 s, 83 °C for 30 s, and 72 °C for 30 s. Three amplification replicates of each sample were performed and then pooled. Step 2 PCR product purifications and quantifications were performed as in Step 1. All barcoded samples were pooled together in equimolar ratio, denatured and sequenced on a Miseq® sequencer (Illumina®). Paired-end sequencing (2 × 250 bp) was performed using MiSeq® reagent Kit V2 (Illumina®). Samples were sequenced in two batches producing a total of 7,962,457 reads with an average of 38,020 reads per samples. Quality (Q) scores were greater than Q30 for 92 and 94% of reads, respectively, for the first and second sequencing batch.

### OTU picking

Using the default parameters of the SmileTrain pipeline (https://github.com/almlab/SmileTrain/wiki), preclustering, quality filtering, primer removal, merging of raw sequences, and postclustering dereplication were performed on raw sequences using USEARCH v. 7.0.1090 [36]. Operational taxonomic units (OTUs) were called from the filtered reads, using a de novo distribution-based clustering method, with *otu_caller.py* script from SmileTrain which performs a custom algorithm using USEARCH. This clustering method takes into account the distribution of DNA sequences across samples and as well as the genetic distance between sequences [37, 38]. Subsequent analyses were performed using QIIME software version 1.8.0 [39]. Using the *assign_taxonomy.py* script, taxonomy was assigned to samples at a 97% identity level with the GreenGenes database version 13_8 [40]. To produce a filtered OTU table, used for the majority of the subsequent analysis, OTUs with fewer than 10 observations across all samples were removed from the OTU table using the *filter_otus_from_otu_table.py* script. In parallel, an unfiltered table including rare OTUs was kept to perform alpha diversity analyses. Each sample was then rarefied to 10,000 reads using the *single_rarefaction.py script*, yielding two rarefied OTU tables, one filtered for rare OTUs and one unfiltered. Twelve samples were eliminated at this stage (< 10,000 reads), including one PCR negative control and five extraction negatives, leaving a total of 172 samples remaining for all downstream analyses. Three extraction and one PCR negatives clustered with microbiome samples in a PCoA ordination, suggesting possible cross-contamination. To check for potential contamination, samples were compared across rows, columns and quadrant of the plate, according to their location in the 96 wells-plates used for library preparation, using the permanova function [41] of the vegan package [42]. No correspondence between negatives and their proximate samples (same row, column, or quadrant) were observed (*p* > 0.05). This suggests that cross-contamination occurred randomly, and likely impacted all samples equally. Because most negative controls contained few reads (< 10,000), we also conclude that cross-contamination had a negligible influence on most samples, and that most reads were not due to contamination.

### Statistical analysis

Except where noted, R software [43] and packages were used for all statistical analyses. A threshold of *α* = 0.05 was considered statistically significant, and *p* values were adjusted with a Bonferroni correction in cases of multiple tests.

To assess alpha diversity within each sample, four metrics were computed from the unfiltered OTU table (i.e., containing rare OTUs with fewer than 10 observations across all samples) using the phyloseq package [44]. Observed OTUs is a metric that counts the number of distinct OTUs in every sample. Chao1 is a non-parametric community richness estimator [45], whereas Shannon and Simpson indices are diversity estimators considering both community richness and evenness. To compare alpha diversity estimates obtained from paired stool and toilet paper samples from the same individual, a paired Student’s *t* test was performed for each metric. To compare alpha diversity estimates across locations, and across samples preservation conditions, Mann–Whitney–Wilcoxon tests were performed for each metric. The Kruskal–Wallis rank sum test for non-parametric data was used to compare alpha diversity across seasons.

To perform beta diversity analysis between samples, distance matrices were computed from the filtered OTU tables to avoid any biases caused by the presence of rare OTUs. Jenson–Shannon divergence (JSD), Bray–Curtis (BC) dissimilarity, unweighted UniFrac, and weighted UniFrac distances were calculated using the phyloseq package. JSD is a metric based on Shannon entropy [46], whereas BC measures the compositional dissimilarity between two samples based on the relative abundance of OTUs. UniFrac uses distances between samples on a phylogenetic tree, either accounting for the relative abundance of OTUs (weighted) or not (unweighted) [47]. Principal coordinate analyses (PCoA) were then performed on the resulting distance matrices using the ggplot2 package [48]. Before computing the ordinations, the Euclidean nature of the distances was verified with the ade4 package [49]. A square-root transformation was applied when the Euclidean property was not respected (which was true for all cases, except for Unweighted UniFrac in Additional file 2 Figures S8A., S9A., and S12A.), ensuring an accurate representation of distances. Sample clustering hypotheses were tested using a permutational multivariate analysis of variance (permanova), and homogeneity of dispersion among sample groups was assessed using the betadisper function [50]. Permanova was selected because it has been shown to be more powerful than other tests to detect differences in community structure, even when group dispersions are heterogeneous [51]. To compare distance matrices computed from toilet paper and stool samples from the same individual, a Mantel [52] test was computed using the ade4 package. All permutations tests were based on 9999 iterations.

Information about food consumption, collected from dietary questionnaires, was compiled in a tabular matrix. Similar food categories were grouped to limit the number of variables and to avoid highly correlated variables (Additional file 1 Table S3). The mean frequency per day (number of times eaten ± standard deviation) of the different food categories was calculated for each sample, from 48 h dietary recall information provided in the questionnaires. Centered and scaled frequencies of consumption of food categories were then used as an explanatory variables in redundancy analyses (RDA) computed on squared-root-transformed unweighted UniFrac distances, using the vegan package. A partial RDA model was then used to estimate the amount of variation in microbiome community composition uniquely explained by diet after controlling for geography (Nunavut or Montréal), or by geography after controlling for diet. Adjusted *R* ^2^ statistics were computed to produce unbiased estimators of explained variation, accounting for the number of predictors in the models.

To compare frequency of consumption of the different food categories across geography, the mean values per individual were calculated for each food categories. Then, we used a permuted Student’s *t* test on each food category to compare geographic locations, with Bonferroni correction for multiple tests. To compare frequency of consumption of the different food categories across season, the mean values per individual were calculated for each category for each season separately. The mean values within each season were compared with a Kruskal–Wallis rank sum test for non-parametric data with Bonferroni correction.

We used linear discriminant analysis (LDA) effect size (LEfSe) [53] to identify differentially abundant taxa between groups of samples [54]. This analysis combines a Kruskal–Wallis test, followed by an LDA step. The subset of OTUs violating the null hypothesis of the Kruskal–Wallis test serves to build the LDA model. From this model, each OTU is assigned an LDA score to assess its association with the categorical variables of interest (e.g., geography, sex).

To investigate the stability of microbiome diversity within individuals over time, we computed the Shannon alpha diversity metric for each sample. Mean alpha diversity values were calculated for each individual. We then defined dispersion as the absolute difference in alpha diversity between each sample from an individual to that individual’s mean alpha diversity value. To compare the two populations (Nunavut vs. Montréal), we pooled the dispersion values separately for individuals from Montréal or Nunavut and used a permutational Student’s *t* test with 9999 permutations to assess the statistical significance of the difference in dispersion. To assess beta-diversity dispersion within each participant, we calculated the distance from each sample to the centroid for that person from unweighted UniFrac distances, using the betadisper function. We pooled the dispersion values separately for individuals from Montréal or Nunavut. To compare the two populations, a permutational Student’s *t* test with 9999 permutations was used to assess the statistical significance of the difference in dispersion.

## Results

### Sampling methods and preservation

**Fig. 1:**
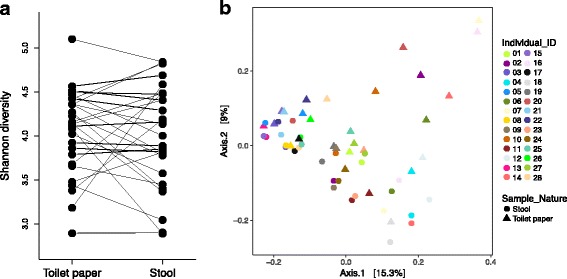
Paired toilet paper and stool samples do not differ in richness, but differ slightly in composition**. a** Comparison of Shannon alpha diversity between paired stool and toilet paper samples. No significant difference in diversity was observed (paired *t* test, *p* = 0.7053). See Additional file 2 Figure S2 for additional alpha diversity measures. **b** Principal coordinates analysis (PCoA) of Unweighted UniFrac distances computed between paired stool and toilet paper samples from the same individual (see Additional file 2 Figure S3 for additional metrics). Each color represents a single individual and the shapes identify the sample type. A small but significant difference was observed between the community compositions of the two sample types (permanova *R* ^2^ = 0.04, *p* = 0.0017) comparatively to a larger significant difference between paired samples (permanova *R* ^2^ = 0.75, *p* = 0.0001)

To compare sampling methods, we obtained and sequenced paired stool and toilet paper samples from the same person at the same point in time (*n* = 28 pairs). We found that stool and toilet paper samples did not differ significantly in OTU richness or diversity (paired Student’s *t* test, *p* > 0.05) (Fig. 1a and Additional file 2 Figure S2). However, we observed small but significant differences in microbial community composition between stool and toilet paper samples, explaining up to 4% of the variation, depending on the beta diversity metric used (Fig. 1b and Additional file 2 Figure S3). Dispersion was not significantly different between toilet paper and stool samples, both with weighted and unweighted UniFrac distances (betadisper, *p* > 0.05) and was significantly different with Bray–Curtis and JSD (respectively *p* = 0.0201 and *p* = 0.0182). Despite slight differences in community composition, most paired stool and toilet paper tended to cluster together in PCoA plots based on unweighted UniFrac distances (Fig. 1b), or other metrics (Additional file 2 Figure S3). A Mantel test comparing unweighted UniFrac distances among individuals based on the paired samples showed higher correlation (*r* = 0.476, *p* = 0.0001) compared to JSD distances (*r* = 0.343, *p* = 0.0380), Bray–Curtis (*r* = 0.297 *p* = 0.0002), or weighted UniFrac distances (*r* = 0.223, *p* = 0.0001) (Additional file 2 Figure S4). Therefore, the community composition of toilet paper samples assessed with unweighted UniFrac is most comparable with stool; thus this measure of beta diversity was used for further analyses of toilet paper samples.

LEfSe identified 27 genera associated with toilet paper, and 10 genera associated with stool samples (LDA score > 2) (Additional file 1 Table S4). From the 27 genera associated with toilet paper, five were also associated with women (Additional file 1 Table S5). Of these five genera, four were found in the top 50 (out of 282) taxa commonly found in a survey of 394 vaginal microbiomes [55]: *Megasphaera* (found in 35% of women), *Aerococcus* (29%), *Gemella* (23%), and *Moryella* (11%), suggesting possible vaginal sampling on toilet paper. However, two genera were associated with both toilet paper and men, and these were also found in vaginal microbiomes: *Prevotella* (found in 69% of women previously studied [55]) and *Sutterella* (8%). *Prevotella* is also found in the gut, and the genus level may lack resolution to distinguish gut vs. vaginal strains. Moreover, *Lactobacillus* dominate the vaginal microbiomes of most women, and this genus is associated with women in our samples (Additional file 1 Table S5), and is approximately 10 times more abundant in women than in men (mean relative abundance of, respectively, 0.0071 and 0.0007). *Lactobacillus* was also 13 times more abundant in toilet paper than stool samples (mean relative abundance of, respectively, 0.0091 and 0.0007) but the difference was not significant by LEfSE analyses (Additional file 1 Table S4). Finally, we observed no significant overlap between toilet paper-associated and women-associated taxa (Fisher’s exact test, *p* = 0.35). This suggests that differences between stool and toilet paper samples are not entirely attributable to sex bias. Overall, these results show that toilet paper provides a similar sampling of the gut microbiome as stool samples, with minor differences due to the sampling of a few skin or vaginal bacteria on toilet paper.

Microbiomes were sampled as consistently as possible in Nunavut and Montréal, but Nunavut toilet paper samples were stored longer at room temperature during shipping. To assess the impact of sample storage on our results, we compared a set of “optimally preserved” toilet paper samples from Nunavut (frozen at − 80 °C within 24 h of sampling, exactly as in Montréal) to “sub-optimal” samples that were stored at room temperature for several days before freezing. We detected no significant differences in alpha diversity between optimal and sub-optimal Nunavut samples (Wilcoxon-test, *p* > 0.05) except for a slight difference in observed OTUs (Wilcoxon, *p* = 0.05). (Additional file 2 Figure S5). Furthermore, no significant effect of preservation methods was observed on community composition (permanova, *p* > 0.05) or dispersion (betadisper, *p* > 0.05) regardless of the beta diversity metric used. These results suggest that subtle differences in sample preservation are unlikely to explain any differences in microbiome diversity or stability between Montréal and Nunavut.

### Individuality, geography and sex shape microbiome composition

**Fig. 2:**
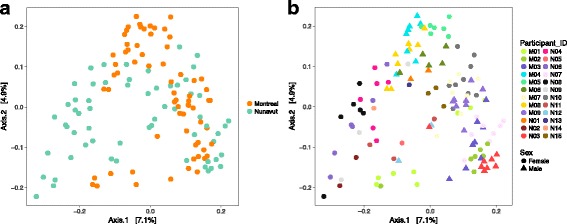
Microbiomes vary mainly by individual, and slightly by geography. Principal coordinates analysis (PCoA) of Unweigthed UniFrac distances computed between paper toilet samples (see Additional file 2 Figure S4 for additional metrics). Montréal and Nunavut microbiomes cluster by (**a**)**.** geography (permanova *R* ^2^ = 0.04, *p* = 0.0001), by (**b**) individual participant identity (permanova *R* ^2^ = 0.45, *p* = 0.0001), and by sex (permanova *R* ^2^ = 0.03, *p* = 0.0001)

Having established that toilet paper and stool samples have similar (but non-identical) microbiome compositions, we proceeded to analyze the remaining 128 toilet paper samples, across 15 individuals from Nunavut and 9 individuals from Montréal (mean of 5 time points per individual). Slightly more OTUs were observed in Montréal compared to Nunavut, but other measures of alpha diversity were similar (Additional file 2 Figure S6), consistent with our previous results [34]. Our previous study did not identify any differences in overall community composition between Nunavut and Montréal, based on a single sample per participant. Using multiple samples over time, we found a modest but significant clustering of microbiomes according to geography (Montréal or Nunavut), explaining 3–5% of variation in community structure, depending on the metric used (Fig. 2a and Additional file 2 Figure S7A). Sex (self-reported male or female) explained about 3–4% of the variation (Fig. 2b and Additional file 2 Figure S7B). Individuality (participant identity) was a much stronger driver of community structure, explaining between 45 and 61% of the data variation, depending on the metric used (Fig. 2b and Additional file 2 Figure S7B). All tests of multivariate dispersion were significant, (betadisper, *p* < 0.05), with greater dispersion in the Nunavut samples compared to Montréal, and in women compared to men. The significantly different dispersions between Nunavut and Montréal indicate that permanova results (Fig. 2) should be interpreted with some caution. For example, the left-hand side of Fig. 2a shows a cluster of participants from Nunavut, which could be explained by higher dispersion among Nunavut samples. We noted that this cluster consisted entirely of women, and therefore asked whether differences in community composition between Nunavut and Montréal could be attributed to confounding sex with geography. To test this, we repeated the beta diversity analyses using samples from women and men separately. In women, we found similar results, with geography explaining 3.5–6.1% of variation in community structure (Additional file 2 Figure S8). In men, geography explains somewhat more (6.7–13.6%) of the variation (Additional file 2 Figure S9). We therefore conclude that Montréal and Nunavut microbiomes differ significantly in their dispersion, and possibly in their community composition. LEfSe revealed 57 taxa more prevalent in Montréal samples, and 57 in Nunavut samples (LDA score > 2) (Additional file 1 Table S6). Most of these taxa were not detected in our previous study [34], suggesting that temporal sampling revealed a larger amount of total microbiome diversity. However, we confirmed three taxa previously associated with Nunavut (*Bacteroidales, Peptococcus*, and TM7) and an additional four specifically associated with a westernized diet in Nunavut (*Prevotella, Sneathia, Actinomyceteaceae*, and *Arcanobacterium*).

### Effects of diet on the microbiome

**Fig. 3:**
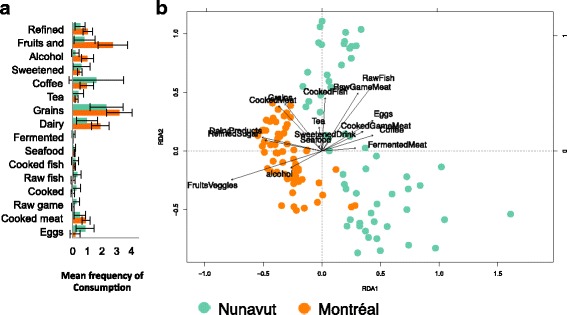
Variation in diet explains differences in microbial community structure between Montréal and Nunavut. **a** Bar chart showing the mean frequency (number of times eaten per participant ± standard deviation) of each food category over the 2 days preceding sampling. "Refined" refers to refined sugars, "Fruits and" refers to fruits and vegetables, and "Sweetened" refers to sweetened drinks. **b** Canonical redundancy analysis (RDA) of Unweigthed UniFrac distances calculated between all toilet paper samples from Montréal and Nunavut, with food groups as explanatory variables (adjusted *R* ^2^ = 0.11, *p* = 0.001). Each point represents a single sample. All time points from all participants are included

Based on dietary surveys, we found significant differences in food consumption frequencies in Montréal and Nunavut. Traditional Inuit food (cooked, raw, and fermented game meats) was exclusively consumed in Nunavut (Fig. 3a). Egg consumption was significantly higher in Nunavut (Permuted *t* test with Bonferroni correction, *p* = 0.0048) and conversely, fruits and vegetables (*p* < 0.0016), alcoholic beverages (*p* = 0.008) and dairy products (*p* = 0.034) were more frequently consumed in Montréal (Fig. 3a). We could not identify any food categories that varied by season in Nunavut (Kruskall–Wallis test, *p* > 0.05 after Bonferroni correction for all food categories; Additional file 2 Figure S10). In Montréal, only egg consumption varied slightly by season (*p* = 0.04; Additional file 2 Figure S10).

To assess the extent of variation in microbiome community composition explained by food, we performed an RDA using the frequency of food categories as explanatory variables. The results of a significant RDA model explaining 11% of community structure variation are illustrated in Fig. 3b. Notably, when RDA clusters microbiome samples by food category, they also cluster according to geography (Fig. 3b), highlighting the contrasting diets of Montréal and Nunavut. For example, raw game meat, raw fish, and coffee appear to be associated with microbiome samples from Nunavut, whereas fruits and vegetables, cooked meat, and alcohol are associated with Montréal (Fig. 3b). These dietary factors explain much of the variation in microbiome composition. Confirming that diet and geography are correlated, a partial RDA found that only 6.5% of variation in community composition could be explained by diet after controlling for geography (adjusted *R* ^2^ = 0.064, *p* = 0.001), and only 3% of the variation could be explained by geography after controlling for diet (adjusted *R* ^2^ = 0.032, *p* = 0.001). The remaining 2% of the variance is explained by the interaction of diet and geography. This suggests that, even though geographical separation has a measurable effect on microbiome composition, the effects of diet are stronger. Consistent with the strong effects of diet, an RDA run on Nunavut samples only showed that diet could explain 17% of variation in community structure (adjusted *R* ^2^ = 0.169, *p* = 0.004).

### Within-individual temporal variation of the Inuit microbiome

**Fig. 4:**
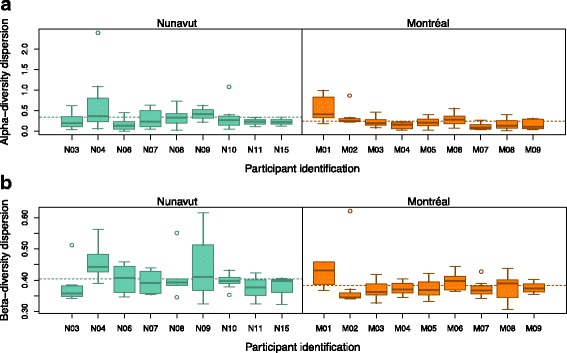
Greater temporal alpha and beta diversity variation within individuals in Nunavut. **a** Distribution of dispersion in alpha diversity over time for each individual participant who contributed at least three samples. Dispersion is here defined as the absolute difference in Shannon diversity in each sample from the mean Shannon diversity for a particular individual. The participants from Nunavut have significantly higher alpha diversity dispersion over time than participants from Montréal (permutational *t* test, *p* = 0.0061). **b** Distribution of dispersion in beta diversity over time within the same participants. Dispersion is here defined as the distance of each sample to the centroid for that individual in compositional space, using unweighted UniFrac distances. The participants from Nunavut have significantly higher dispersion in beta diversity over time than participants from Montréal (permutational *t* test, *p* = 0.0124)

We expected that the Inuit microbiome would vary seasonally across 8 months of sampling, while the Montréal microbiome would not (because commercial foods are available all year long). Contrary to this expectation, we observed no significant difference in alpha diversity over seasons in either Nunavut or Montréal (Kruskal-Wallis, *p* > 0.05; Additional file 2 Figure S11). No significant clustering of microbiomes according to season (or month) was observed in either Nunavut or Montréal (permanova, *p* > 0.05; Additional file 2 Figure S12), and no particular taxa were significantly associated with seasons in either Montréal or Nunavut (LEfSE, *p* > 0.05). However, multivariate dispersion among months and seasons were similar in Montréal (betadisper, *p* > 0.05), but not in Nunavut (betadisper, *p* < 0.05). To further investigate this apparent difference in temporal variability between Nunavut and Montréal, we estimated the dispersion of both alpha diversity and beta diversity (community composition) within each individual over time (Fig. 4). These analyses show significantly higher dispersion within individuals in Nunavut compared to Montréal. In particular, a few individuals in Nunavut (N04 and N09) have highly variable alpha and beta diversity over time (Fig. 4). Some of these temporal variations could be explained by baseline levels of alpha diversity, as previously shown in American college students [12]. Consistently, we found that individuals with higher median alpha diversity tended to have less temporal variation in community composition (Spearman rank correlation between median Shannon diversity and median unweighted UniFrac distance over time, rho = −0.4283, *p* = 0.078; Additional file 2 Figure S13).

## Discussion

### Stool and toilet paper provide similar but non-identical portraits of the microbiome

Sample collection in a remote community, with no field coordinator on location, forced us to use strategies to facilitate collection and shipping of samples. Consequently, we collected mainly toilet paper containing stool, instead of complete stool, to sample the gut microbiome. Although this method has been used in previous studies [12, 56], differences between stool and toilet paper samples have not previously been assessed. Our comparative analyses show that toilet paper and stool samples provide similar estimates of alpha and beta diversity, with up to 4% of the community composition variation explained by sampling method. Therefore, toilet paper provides a fairly similar picture of the gut microbiome as stool, with some contribution (~4%) of the skin and urogenital microbiomes.

Regarding sample preservation, our results are consistent with previous studies showing that storing stool samples at ambient temperature for weeks did not dramatically alter the microbiome [57–59]. Toilet paper samples are dryer and less concentrated than complete stool samples. We speculate that these characteristics might make toilet paper samples less susceptible to change in microbiome composition during storage, compared to whole stool.

### The traditional Inuit diet influences microbiome composition

The diversity and composition of the human gut microbiome varies widely by geography, mainly due to differences in diet [9, 18, 19, 60]. While most ‘traditional’ diets studied to date are high in fiber and high in microbiome diversity, the traditional Inuit diet is low in fiber, and is associated with similar levels of microbiome diversity as the Western diet [34]. Here, we extended the study of the Inuit microbiome over many months of sampling. We confirmed the similar levels of diversity between Inuit and urbanized Western microbiomes, but our time-series sampling allowed us to detect differences in community composition that were mostly attributable to differences in diet. Specifically, dietary choices explained 17% of the variation in microbiome community composition in Nunavut, with traditional foods such as raw fish, raw game meat, and fermented meats as major drivers (Fig. 3b). This suggests that, in spite of a transition to an increasingly Western diet [26], the traditional Inuit diet still has an impact on the microbiome.

### Temporal variation of the Inuit microbiome

Previous studies of temporal variations in the microbiome have shown that within-individual differences are usually less pronounced than among-individuals differences [61, 62]. Our results confirm this observation, with participant identity explaining up to 61% of variation in community composition. Nevertheless, some individuals were more variable over time than others (Fig. 4), supporting the idea that within-individual microbiome variation is a personalized feature, as suggested in another time-course study [12]. This previous study also found that highly diverse gut microbiomes tend to maintain more stable community compositions over time, a trend we also observed in our study participants (Additional file 2 Figure S13).

We hypothesized that the Inuit microbiome would vary over time, according to seasonal food availability. However, we found no evidence for a shift in microbiome composition with month or season (Additional file 2 Figure S12) in either Montréal or Nunavut samples. A previous study of a traditional Hutterites community showed a significant shift in the gut microbiome composition between winter and summer, due to seasonal changes in diet [20]. The Hutterites consumed more fruits and vegetables in the summer compared to the winter. In contrast, we did not detect any significant seasonal differences in diet in our Inuit participants (Additional file 2 Figure S10). It is likely that seasonal differences in diet do exist (as previously documented [31, 32] but were not captured in our sample, or at the resolution of our dietary questionnaire. Even if seasonal dietary differences do exist, they are apparently not strong enough to produce a systematic shift in the composition of the average Inuit microbiome from season to season, or from month to month (Additional file 2 Figure S12).

We also found that microbiome community dispersion did not vary seasonally in Montréal (betadisper, *p* > 0.05), but did vary in Nunavut (betadisper, *p* < 0.05). This suggests that certain seasons or months lead to higher among- and within-individual variation in microbiome compositions, although the community does not change in a particular direction, and is not consistent across individuals (Additional file 2 Figure S12). This suggests that there are certain times of year when diet (or other unmeasured lifestyle factors) is more variable in Nunavut, but not in Montréal—perhaps due to greater food insecurity in Nunavut. This could explain why Nunavut microbiomes varied significantly more over time than in Montréal, both in terms of alpha diversity and beta diversity (Fig. 4). The higher temporal variability in Nunavut could be explained by the higher mean age of our Nunavut participants. However, the individuals with the highest variability in alpha and beta diversity over time (Fig. 4) had a range of ages: N04 (60 years old), N09 (26 years), M01 (23 years). This suggests that, in our dataset, age is not a clear determinant of microbiome variability over time. Part of this variability could be due to opportunistic and highly individual hunting and gathering practices, and part could be due to variability in commercial food supply, which also varies according to shipping conditions and food prices [29]. Food sharing between individuals and families is a popular practice in Inuit communities [29]. If such sharing occurred at the level of the entire community, this might homogenize microbiomes across individuals at certain times of year – a trend which is not observed (Additional file 2 Figure S12). Therefore, sharing might only occur within certain groups or families within the community, obscuring directional seasonal shifts in diet or the microbiome. In contrast, most products are available in Montréal yearlong, allowing people to consume a diversity of foods regardless of season. Consequently, food categories can be consumed homogenously across time. Taken together, such differences in availability, diversity, and quality of food in the North affect the traditional Inuit diet, which in turn impacts the diversity and stability of the gut microbiome over time.

## Conclusion

Sampling the Inuit microbiome over time allowed us to capture variation in community composition that was not evident from a single time point. We found that dietary choices, including traditional foods such as raw fish and fermented meats, have a significant impact on community composition. The Inuit microbiome lacked the expected seasonality, perhaps due to an increasingly westernized diet. Despite the lack of directional seasonal shifts in composition, Inuit microbiomes are more variable over time, in both diversity and composition, than their urbanized counterparts. This might be due to opportunistic and individualized food choices, due both to hunting practices and to fluctuations in the prices of imported foods. From a methodological perspective, our study showed that toilet paper sampling is fairly representative of stool, providing a simple and convenient alternative to whole stool sampling and shipping. Overall, our study highlights the importance of time-series data to capture the total diversity of the human microbiome.

## Additional files

Additional file 1: Supplementary Tables S1 to S6. (XLSX 29 kb)

Additional file 2: Supplementary Figures. S1 to S13. (DOCX 9637 kb)

Additional file 3: Dietary habit questionnaire containing information about food consumption in the 48 h preceding the sampling event. (PDF 300 kb)

## Abbreviations

JSD: Jenson-Shannon divergence
LDA: Linear discriminant analysis
LEfSe: Linear discriminant analysis effect size
OTU: Operational taxonomic unit
*p*: *P* value (probability value)
PCoA: Principal coordinates analysis
PCR: Polymerase chain reaction
Permanova: Permutational multivariate analysis of variance
R^2^: Coefficient of determination
RDA: redundancy analysis
