# Supplementary material for: The Inuit gut microbiome is dynamic over time and shaped by traditional foods
Source: Microbiome. 2017 Nov 16;5:151. doi: 10.1186/s40168-017-0370-7 (PMC5689144; doi:10.1186/s40168-017-0370-7)
Supplement: Supplementary file 2 — Supplementary Figures. S1 to S13. (DOCX 9637 kb) [file 40168_2017_370_MOESM2_ESM.docx]

**A.**

**
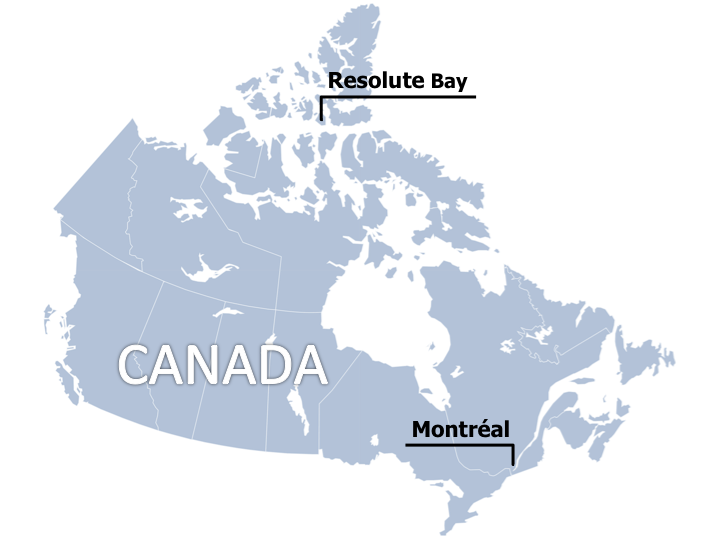
**

**B. C. D.**

**
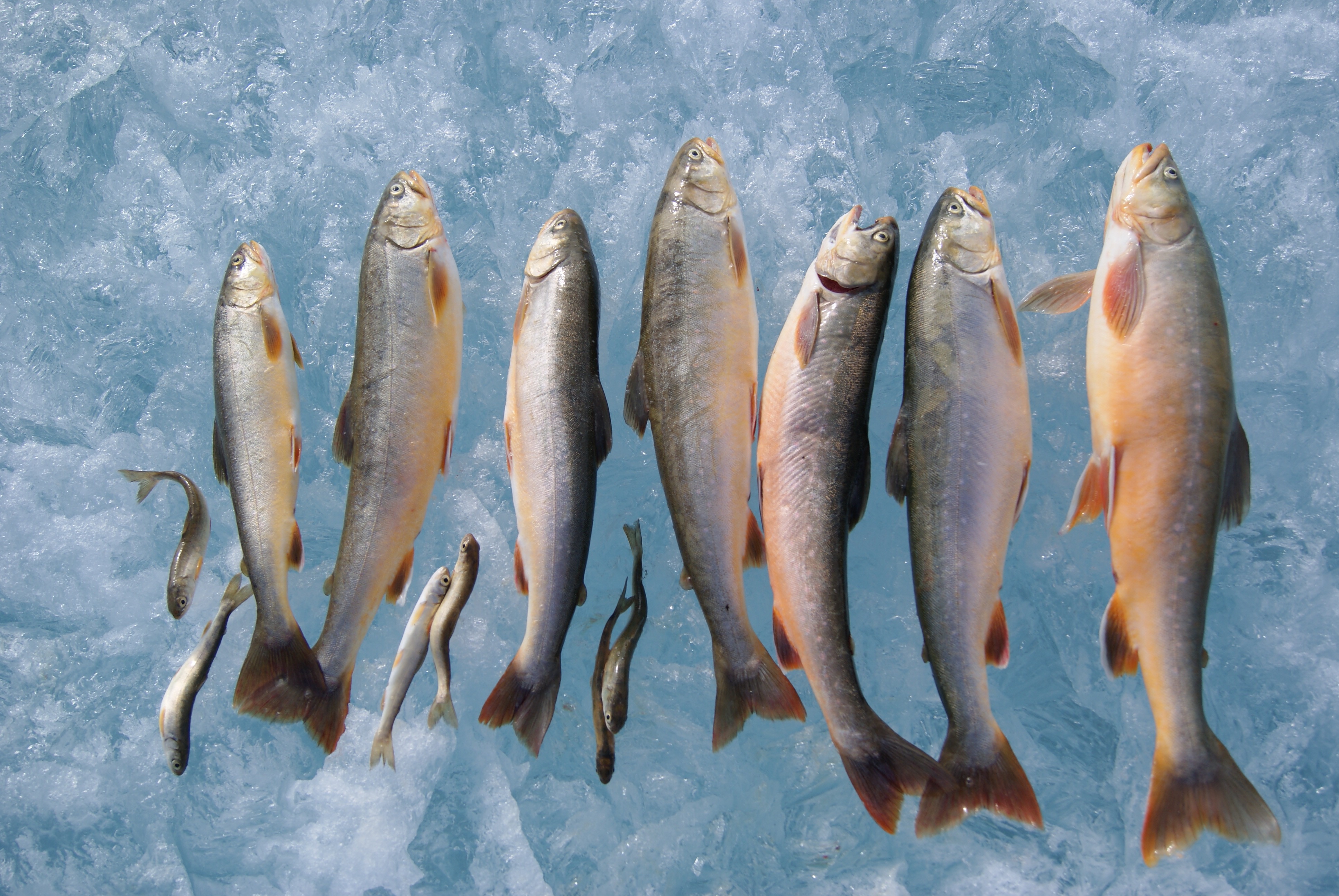

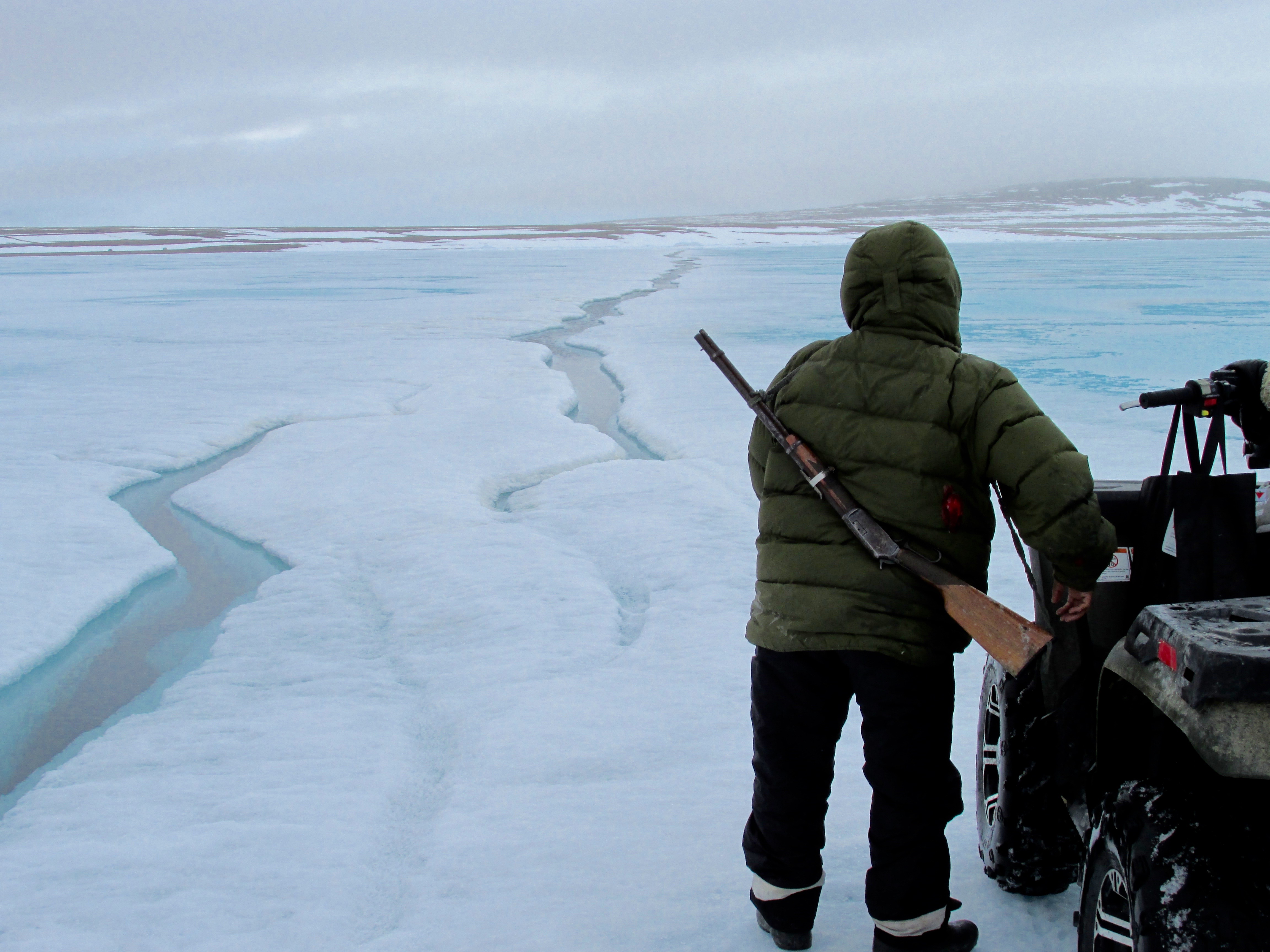

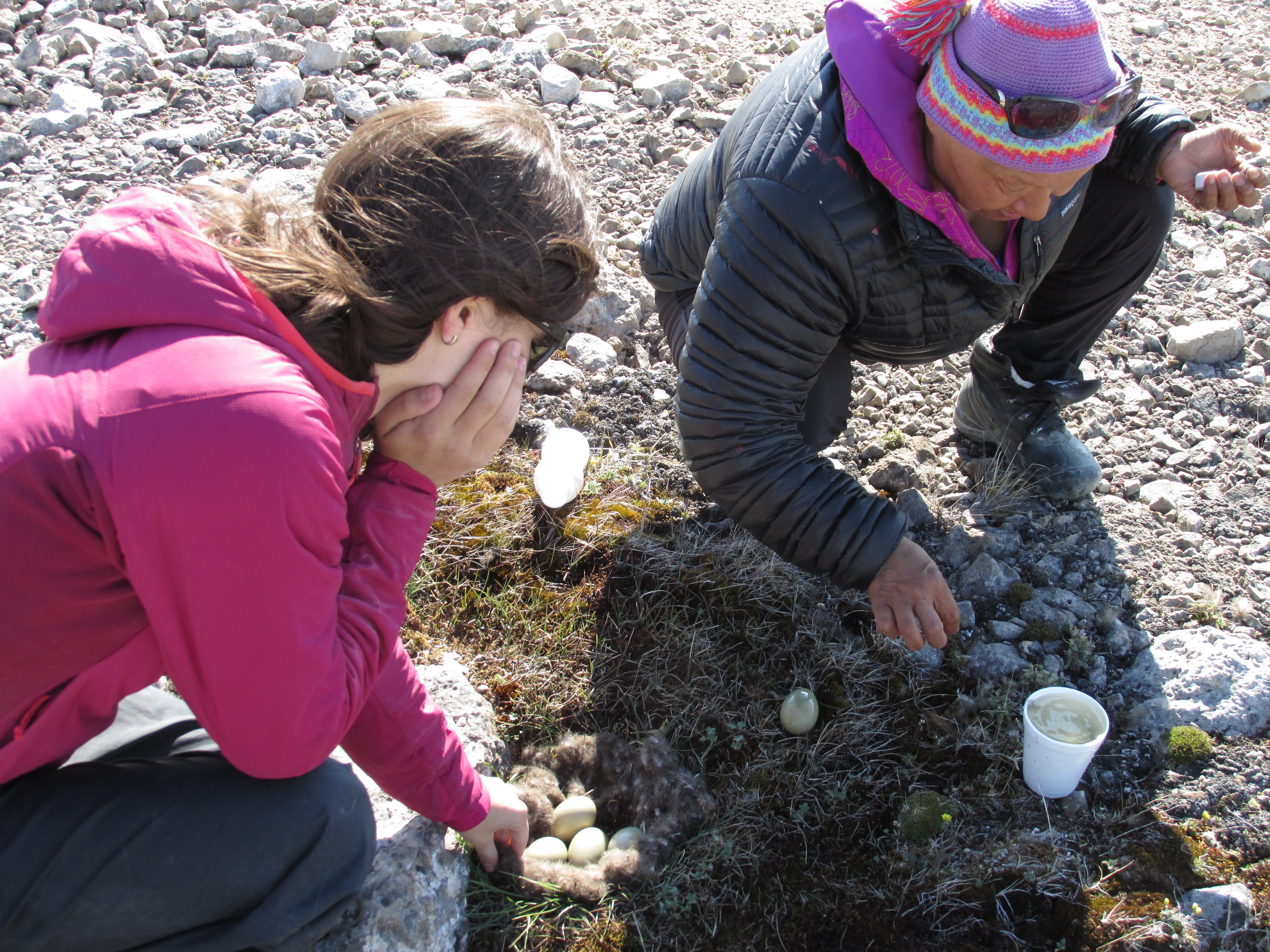
**

**Figure S1. Sampling locations in Nunavut and Montréal and examples of Inuit hunting practices.**

1. Location of Resolute Bay (Nunavut, Canada) and Montréal (Québec, Canada)
2. Freshly caught Arctic char
3. Seal hunting during summer
4. Gathering of wild bird eggs

**A. B. C.**

**Figure S2. Comparison of alpha diversity estimates from the same individual at the same time point.** No significant difference between methods were observed between paired stool and toilet paper samples in **A**. the number of observed OTUs (paired t-test, *p*= 0.3726), **B**. the Chao1 diversity index (paired t-test, *p* = 0.3187), or **C**. the Simpson diversity index (paired t-test, *p* = 0.2915).

**A.** **Weighted UniFrac B. J.-S. divergence C. Bray-Curtis**

Individual ID: R^2^ = 0.635, *p* = 0.0001 Individual ID: R^2^ = 0.766, *p* = 0.0001 Individual ID: R^2^ = 0.688, *p* = 0.0001 Sample Nature: R^2^ = 0.007, *p* = 0.0181 Sample Nature: R^2^ = 0.026, *p* = 0.0464 Sample Nature: R^2^ = 0.022, *p* = 0.0888

**Figure S3.** **Comparison of beta diversity between paired stool and toilet paper samples.** Principal coordinates analysis (PCoA) of **A.** weighted UniFrac distances, **B.** Jenson-Shannon divergence and **C.** Bray-Curtis computed between paired stool and toilet paper samples from the same individual at the same time point. The different colors represent sample pairs (Individual ID) and the shapes indicate sample types (Sample Nature). Results of permanova tests are provided for each comparison.

**A.** **Unweighted UniFrac B. Weighted UniFrac**  Mantel test : 0.477 Mantel test : 0.223

*p* = 0.0001 *p* = 0.038


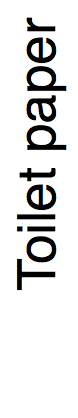

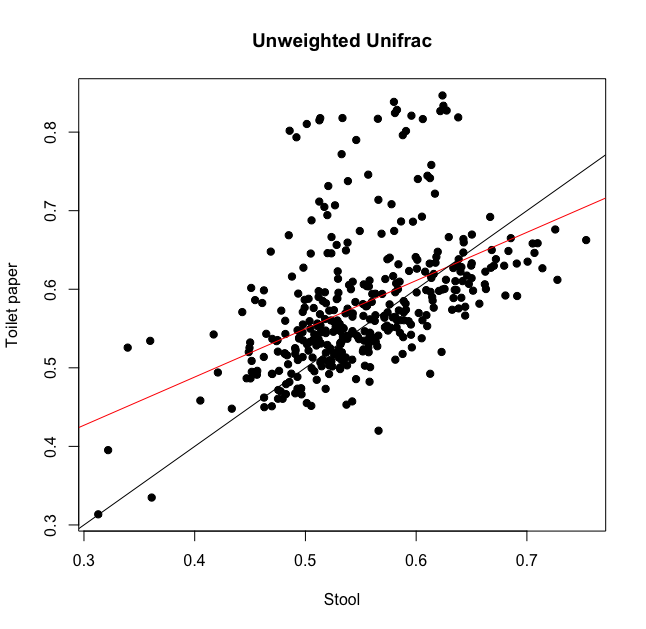

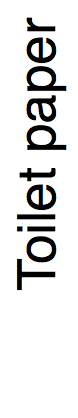

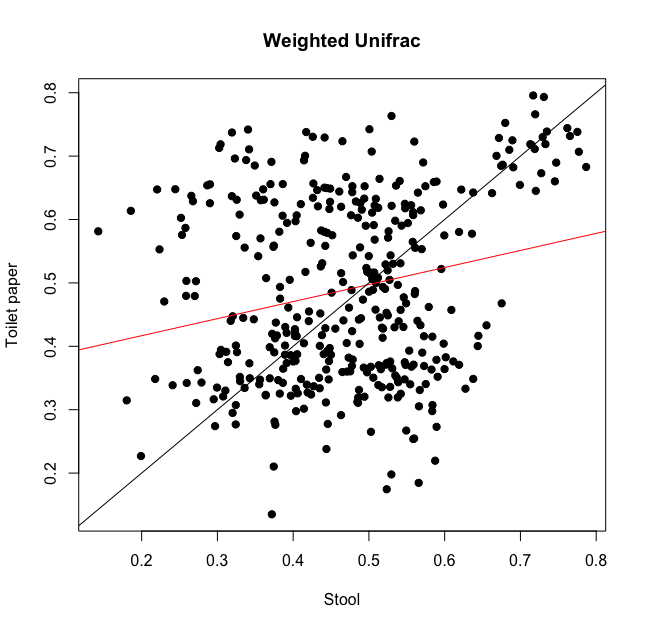


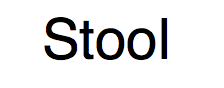

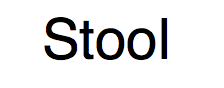


**C. Jenson-Shannon Divergence**  **D. Bray-Curtis Dissimilarity**

Mantel test : 0.343 Mantel test : 0.297

*p* = 0.0001 *p* = 0.0002


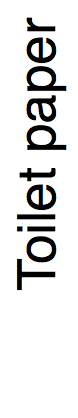
**
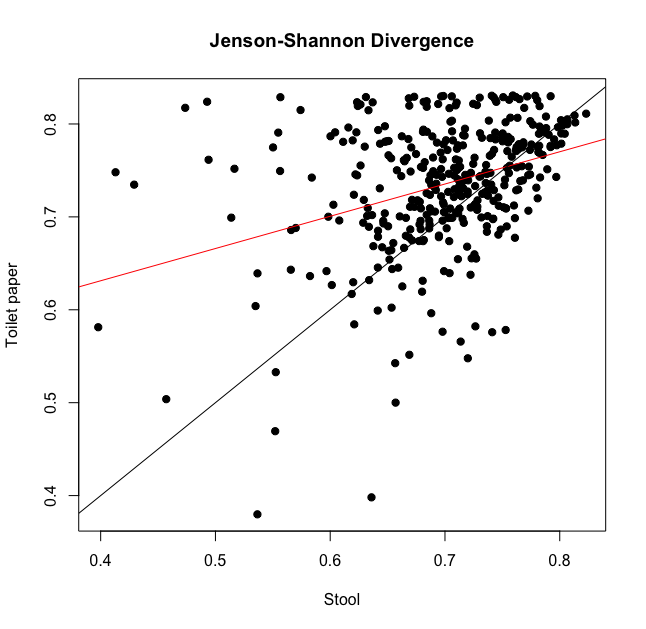
**
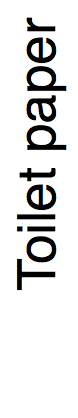
 ****


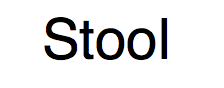

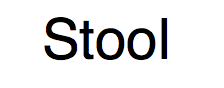


**Figure S4.** **Unweighted UniFrac offers a comparable portrait of microbiome beta diversity between toilet paper and stool.** Scatter plot of distances between paired samples obtained with stool and toilet paper for **A.** unweighted UniFrac distances, **B.** weighted UniFrac distances, **C.** Jenson-Shannon divergence and **D.** Bray-Curtis dissimilarity Results of Mantel tests are provided for each metric.


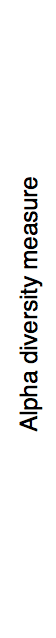


**Figure S5.** **Comparison of diversity between optimal and sub-optimally preserved samples.** Alpha diversity comparisons between sub-optimal and optimal preservation of the toilet paper samples, using four different metrics. All comparisons are not significantly different (Wilcoxon, *p* > 0.05), except observed OTUs (Wilcoxon, *p* = 0.05).


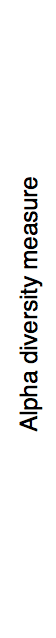


**Figure S6. Similar levels of richness and diversity are observed in Nunavut and Montréal samples.** Comparison of alpha diversity value distributions across Montréal and Nunavut samples, using observed OTUs, Chao1, Shannon, and Simpson indices. Observed OTUs is the only measure revealing significant differences between locations (Wilcoxon-test, *p* = 0.0179).

**A. Geography**

**Weighted UniFrac Jenson-Shannon divergence Bray-Curtis** R^2^ = 0.033, *p* = 0.0003 R^2^ = 0.046, *p* = 0.0001 R^2^ = 0.033, *p* = 0.0001

**B. Participant and Sex**

**Weighted UniFrac** **Jenson-Shannon divergence** Bray-Curtis Participant: R^2^ = 0.548, *p* = 0.0001 Participant: R^2^ = 0.605, *p* = 0.0001 Participant: R^2^ = 0.478, *p* = 0.0001 Sex: R^2^ = 0.037, *p* = 0.0001 Sex: R^2^ = 0.042, *p* = 0.0001 Sex: R^2^ = 0.031, *p* = 0.0001

**Figure S7.** **Geography, participants and sex viewed with additional distance metrics.** Principal coordinates analysis (PCoA) of weighted UniFrac distances, Jenson-Shannon divergence and Bray-Curtis dissimilarity computed among paper toilet samples. Montréal and Nunavut gut microbiomes cluster by **A.** geography, and by **B.** participants and by sex. Results of permanova tests are provided for each comparison. Multivariate dispersions among groups (geography, sex, or participant) were all significantly different (betadisper, *p* < 0.05).

**A. Unweighted UniFrac B. Weighted UniFrac**

R^2^ = 0.0479, *p* =0.0001 R^2^ = 0.0352, *p* = 0.0006

**C. Jenson-Shannon Divergence D. Bray-Curtis**

R^2^ = 0.0613, *p* =0.0001 R^2^ = 0.0462, *p* = 0.0001

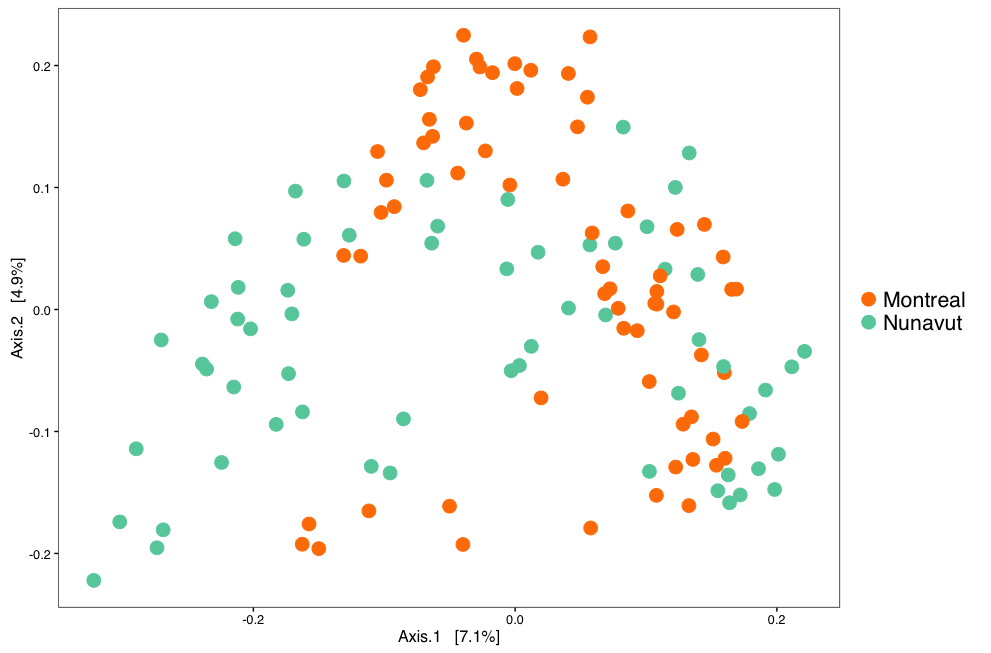
 Nunavut
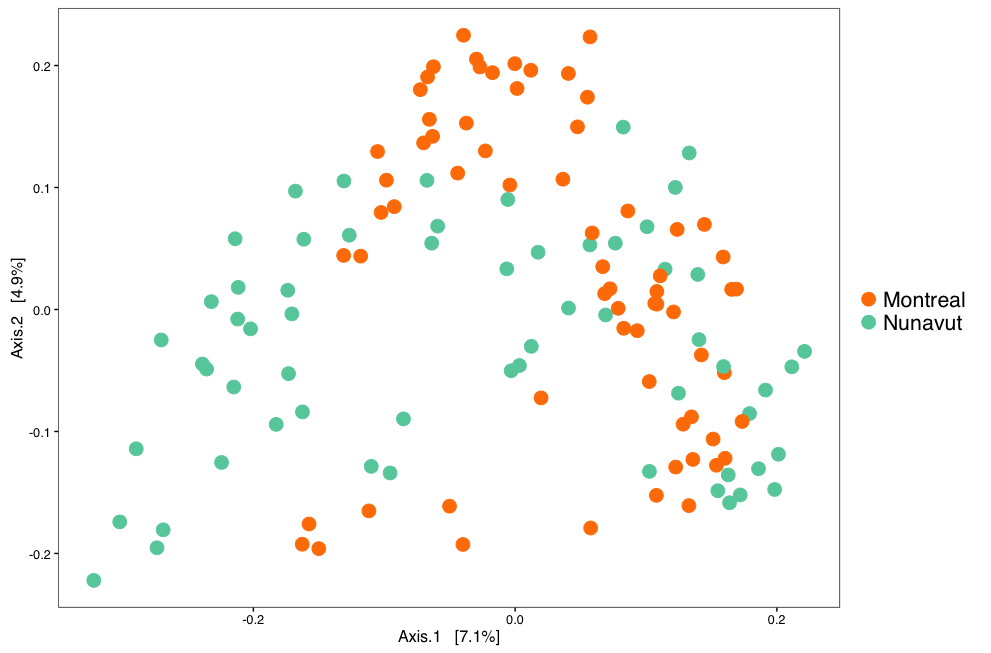
 Montréal

**Figure S8. Samples cluster by geography, only including samples from women.** Principal coordinates analysis (PCoA) of **A.** unweighted UniFrac, **B.** weighted UniFrac, **C.** Jenson-Shannon divergence and **D.** Bray-Curtis dissimilarity computed between paper toilet samples from Nunavut and Montréal, using only samples from women. Permanova *p*-values and R^2^ are provided for each metric. Multivariate dispersions between geographic regions (Montréal or Nunavut) were all significantly different (betadisper, *p* < 0.05) except for Weighted UniFrac (*p* = 0.0914).

**A. Unweighted UniFrac B. Weighted UniFrac**

R^2^ = 0.1364, *p* =0.0001 R^2^ = 0.0669, *p* = 0.0001

**C. Jenson-Shannon Divergence D. Bray-Curtis**

R^2^ = 0.1268, *p* =0.0001 R^2^ = 0.0905, *p* = 0.0001

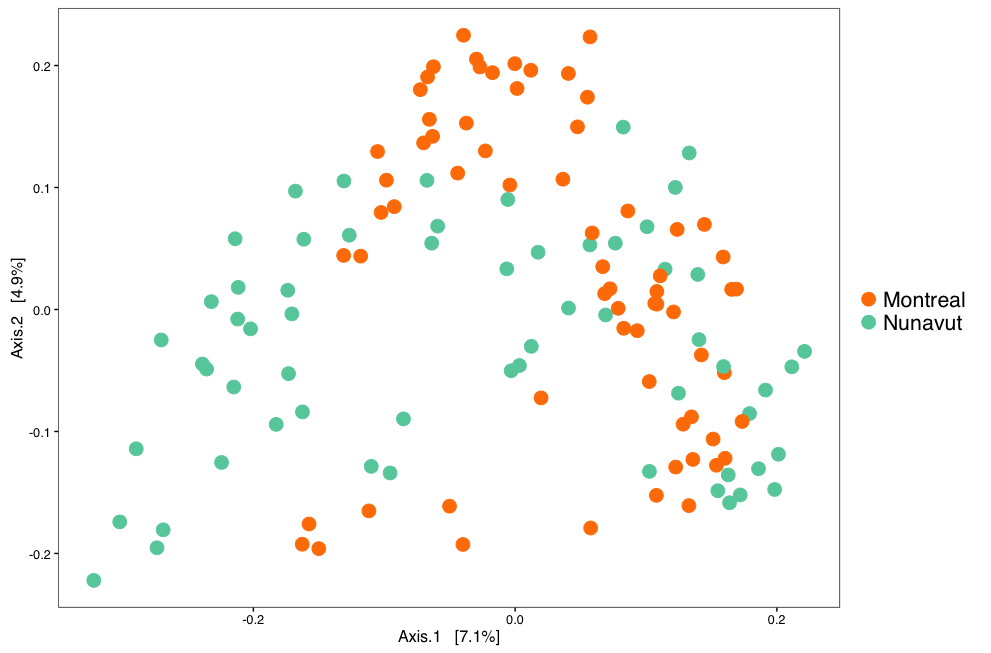
 Nunavut
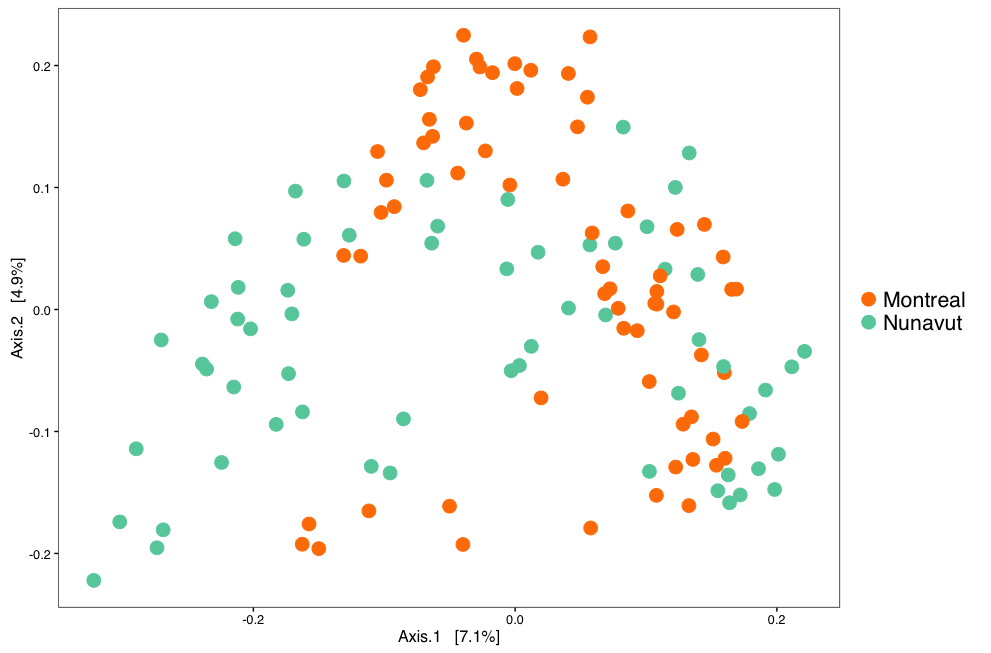
 Montréal

**Figure S9. Samples cluster by geography, only including samples from men.** Principal coordinates analysis (PCoA) of **A.** unweighted UniFrac, **B.** weighted UniFrac, **C.** Jenson-Shannon divergence and **D.** Bray-Curtis dissimilarity computed between paper toilet samples from Nunavut and Montréal, using only samples from men. Permanova *p*-values and R^2^ are provided for each metric. Multivariate dispersions between geographic regions (Montréal or Nunavut) were significantly different (betadisper, *p* < 0.05) except for Unweighted and Weighted UniFrac (*p* = 0.2878 and *p* = 0.1615).

**A. Nunavut**

**B. Montréal**

**Figure S10.** Food categories broken down according to seasons in **A.** Nunavut and **B.** Montréal.

**A.** **Nunavut**

**B.** **Montréal**

**Figure S11: No significant differences in diversity observed among seasons in either Nunavut or Montréal microbiomes.** Boxplots show the distribution of alpha diversity values across Montréal and Nunavut samples according to seasons, using observed OTUs, Chao1, Shannon, and Simpson indices. We observed no significant differences between seasons in either location (Kruskal-Wallis rank sum test , *p* > 0.05)

**A.** **Nunavut** Months: *p* = 0.9906 Months: *p* = 0.9716 Months: *p* = 0.9999 Months: *p* = 0.9999 Seasons: *p* = 0.9777 Seasons: *p* = 0.7526 Seasons: *p* = 0.9395 Seasons: *p* = 0.9271

**B.** **Montréal**

Months: *p* = 1.0000 Months: *p* = 1.0000 Months: *p* = 1.0000 Months: *p* = 1.0000 Seasons: *p* = 0.9686 Seasons: *p* = 0.6165 Seasons: *p* = 0.9232 Seasons: *p* = 0.9107

**Unweighted UniFrac Weighted UniFrac JSD Bray-Curtis**

**Figure S12.** **No clear temporal or seasonal clustering of microbiomes in either Nunavut or Montréal.** Principal coordinates analysis (PCoA) of unweighted UniFrac, weighted UniFrac, Jenson-Shannon divergence and Bray-Curtis dissimilarity (from left to right) computed between paper toilet samples from **A.** Nunavut and **B.** Montréal. Permanova *p*-values are provided for each metric. Multivariate dispersions among group (seasons, months) were significantly different (betadisper, *p* < 0.05) in Nunavut except for seasons with Unweighted and Weighted UniFrac (*p* = 0.2152 and *p* = 0.0638). Multivariate dispersions among group (seasons, months) were all not significantly different (betadisper, *p* > 0.05) in Montréal.

**Figure S13.** **Highly diverse microbiomes tend to be more stable through time.** Spearman rank correlation between the median unweighted Unifrac distance for each participant and the median Shannon Index (Rho = -0.4283, *p* = 0.078). Each point represents a single study participant.
