# Supplementary material for: The Inuit gut microbiome is dynamic over time and shaped by traditional foods
Source: Microbiome. 2017 Nov 16;5:151. doi: 10.1186/s40168-017-0370-7 (PMC5689144; doi:10.1186/s40168-017-0370-7)

DIETARY HABIT QUESTIONNAIRE

Project title
Temporal variation of the Inuit gut microflora in response to seasonal dietary changes

Researchers

- Geneviève Dubois: M. Sc. candidate, fieldwork coordinator
    - Department of biological sciences, Université de Montréal
      Adress :
    - Telephone: Fax:

- Catherine Girard: Ph. D. candidate, associate researcher
    - Department of biological sciences, Université de Montréal
      Adress :
    - Telephone: Fax:

Information on participant

Participant number:

Participant age:

Health state

1. In the last month, have you been sick (diarrhea, vomiting)?
   Yes □ No □

2. In the last month, did you take any antibiotics?
   Yes □ No □

3. In the last month, have you been taking any prescription drugs?
   Yes □ No □

4. In the last month, did you become you pregnant?
   Yes □ No □

5. In the last month, did you travel to another community?
   Yes □ No □

Monthly diet

This section concerns your food consumption in the **last month**.

1. What kinds of meat, fish or seafood did you eat **most often** during the last month? :
____

Daily diet

This section concerns your food consumption in the **last two days**.

1. Meat, fish, and seafood

In the last two days, did you eat **meat**, **fish** or **seafood**? Yes □ No □

If you answered yes, check the boxes indicating what you ate, how you ate it, and indicate how many meals:

□ Chicken	Cooked □	Raw □	How many meals _____
□ Turkey	Cooked □	Raw □	How many meals _____
□ Goose or duck	Cooked □	Raw □	How many meals _____
□ Ptarmigan	Cooked □	Raw □	How many meals _____
□ Eggs	Cooked □	Raw □	How many meals _____
□ Polar bear	Cooked □	Raw □	How many meals _____
□ Muskox	Cooked □	Raw □	How many meals _____
□ Caribou	Cooked □	Raw □	How many meals _____
□ Arctic Hare	Cooked □	Raw □	How many meals _____
□ Seal	Cooked □	Raw □	How many meals _____
□ Walrus	Cooked □	Raw □	How many meals _____
□ Whale	Cooked □	Raw □	How many meals _____
□ Cow meat (steak, hamburger, etc.)	Cooked □	Raw □	How many meals _____
□ Pork meat (bacon, ham, chops, etc.)	Cooked □	Raw □	How many meals _____
□ Other mammals:_________________	Cooked □	Raw □	How many meals _____
□ Market fish	Cooked □	Raw □	How many meals _____
□ Arctic char	Cooked □	Raw □	How many meals _____
□ Other fishes (caught in Resolute Bay):__________	Cooked □	Raw □	How many meals _____
□ Oysters, mussels, scallops	Cooked □	Raw □	How many meals _____
□ Other seafood (shrimps, crab, etc.)	Cooked □	Raw □	How many meals _____
□ Fermented meat (walrus,caribou,etc.)	How many meals _____		

3. Dairy products

In the last two days, did you eat **dairy products**? Yes □ No □

If you answered yes, check the boxes indicating what you ate, and indicate approximately how many meals:

Cheese	□	How many meals ____
Yogurt	□	How many meals ____
Evaporated milk (canned)	□	How many meals ____
Milk	□	How many meals ____
Ice cream	□	How many meals ____

4. Grains

In the last two days, did you eat **grains**? Yes □ No □

If you answered yes, check the boxes indicating what you ate, and indicate approximately how many meals:

Cereal	□	How many meals ____
Bannock	□	How many meals ____
Oatmeal	□	How many meals ____
Brown rice	□	How many meals ____
Brown bread	□	How many meals ____
White rice	□	How many meals ____
White bread	□	How many meals ____
Pasta	□	How many meals ____

5. Beverages

In the last two days, what **beverages did you drink?**

Check the boxes indicating what you drank, and indicate approximately how many glasses or cups:

Green tea	□	How many glasses/cups____
Black tea	□	How many glasses/cups____
Coffee	□	How many glasses/cups____
Milk	□	How many glasses/cups____
Soft drink	□	How many glasses/cups____
Fruit juice	□	How many glasses/cups____
Alcohol	□	How many glasses/cups ____
Snow water	□	How many glasses/cups____
Tap water	□	How many glasses/cups____

6. Fruits and vegetables

In the last two days, did you eat **Fruits** and **vegetables**? Yes □ No □

If you answered yes, indicate approximately how many meals included fruits or vegetables__________

7. Others

In the last two days, did you eat **sweets** or **salty snacks**? Yes □ No □

If you answered yes, check the boxes indicating what you ate:

Candy, chocolate □
Chips, crisps, cheese puffs □
Donuts, cake, cookies □
Others:__________ □

Stool consistency

Circle the picture that looks most like your stool.

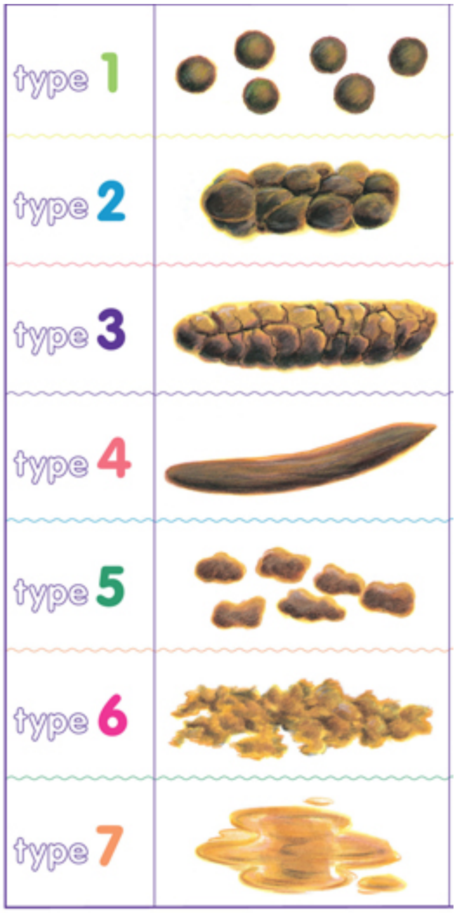

Supplement: Supplementary file 3 — Dietary habit questionnaire containing information about food consumption in the 48 h preceding the sampling event. (PDF 300 kb) [file 40168_2017_370_MOESM3_ESM.pdf]
